# RE-AIM in Clinical, Community, and Corporate Settings: Perspectives, Strategies, and Recommendations to Enhance Public Health Impact

**DOI:** 10.3389/fpubh.2018.00071

**Published:** 2018-03-22

**Authors:** Samantha M. Harden, Matthew Lee Smith, Marcia G. Ory, Renae L. Smith-Ray, Paul A. Estabrooks, Russell E. Glasgow

**Affiliations:** ^1^Physical Activity Research and Community Implementation, Human Nutrition, Foods, and Exercise, Virginia Tech, Blacksburg, VA, United States; ^2^Center for Population Health and Management, Texas A&M University, College Station, TX, United States; ^3^Department of Environmental and Occupational Health, School of Public Health, Texas A&M University, College Station, TX, United States; ^4^Department of Health Promotion and Behavior, College of Public Health, The University of Georgia, Athens, GA, United States; ^5^Walgreens Center for Health and Wellbeing Research, Deerfield, IL, United States; ^6^Department of Health Promotion, College of Public Health, University of Nebraska Medical Center, Omaha, NE, United States; ^7^Department of Family Medicine, School of Medicine, University of Colorado, Aurora, IL, United States

**Keywords:** translation, health promotion, knowledge transfer, implementation science, evaluation framework, dissemination and implementation research

## Abstract

The RE-AIM Framework is a planning and evaluation model that has been used in a variety of settings to address various programmatic, environmental, and policy innovations for improving population health. In addition to the broad application and diverse use of the framework, there are lessons learned and recommendations for the future use of the framework across clinical, community, and corporate settings. The purposes of this article are to: (A) provide a brief overview of the RE-AIM Framework and its pragmatic use for planning and evaluation; (B) offer recommendations to facilitate the application of RE-AIM in clinical, community, and corporate settings; and (C) share perspectives and lessons learned about employing RE-AIM dimensions in the planning, implementation, and evaluation phases within these different settings. In this article, we demonstrate how the RE-AIM concepts and elements within each dimension can be applied by researchers and practitioners in diverse settings, among diverse populations and for diverse health topics.

## Introduction

Dissemination and implementation (D&I) research addresses the “how and why” related to strategies for information sharing (dissemination) and intervention integration (implementation) for the purposes of enhancing evidence-based program delivery and population health (1–5). The advancement of D&I science requires a focus on the wide-scale adoption, implementation, and generalizability of program and policy impacts. With well over 100 different models and frameworks utilized in the field (6), researchers and practitioners can become overwhelmed when selecting (and attempting to apply) the most appropriate model/framework for their scientific inquiry or initiative.^1^

The purposes of this article are to: (A) provide a brief overview of the RE-AIM Framework and its pragmatic use for planning and evaluation; (B) offer recommendations to facilitate the application of RE-AIM in clinical, community, and corporate settings; and (C) share perspectives and lessons learned about employing RE-AIM elements in the planning, implementation, and evaluation phases within these different settings. In this article, we demonstrate how RE-AIM concepts and elements can be applied by researchers and practitioners in diverse settings, among diverse populations, and for diverse health topics.

## The RE-AIM Framework

The RE-AIM Framework (7, 8) is often used in D&I research (9, 10), which encompasses essential translational research elements. RE-AIM was identified as the most frequently used model or framework between 2000 and 2016 for D&I grant applications submitted to the National Institutes of Health and Centers for Disease Control and Prevention (CDC) (11). This widespread use is, in part, due to the flexibility to address different public health concerns in a practical manner understandable by practitioners and policy makers. The acronym RE-AIM stands for *r*each (How do I reach those who need a specific intervention?), *e*fficacy/*e*ffectiveness (How do I know my intervention is working?), *a*doption (How do I design for dissemination and develop organizational support to deliver my intervention?), *i*mplementation (How do I ensure the intervention is feasible and delivered properly?), and *m*aintenance (How do I ensure long-term benefits and institutionalization of the intervention and continued community capacity for D&I?).

Applying RE-AIM challenges researchers and practitioners to ask fundamental questions about complex issues before, during, and after the implementation of a putative program in “real world” settings. Among the many strengths of RE-AIM is its robust structure that facilitates broad use across settings (e.g., organization, regional, rural), populations (e.g., age, race/ethnicity, occupation/role), topics (e.g., disease, behavior), and interventions (e.g., demonstration, experimental, translational, longitudinal, multi-level). While the basic RE-AIM dimensions have remained constant since its development in the 1990s (7), its use has evolved over time with new applications in clinical (12), community (13), and corporate (14) settings. A recent systematic review (15) reported health-care (49%) and community (46%) settings applied RE-AIM in empirical or evaluative interventions most frequently; however, no such interventions were reported in corporate settings. As such, efforts are needed to understand the use of RE-AIM in multiple settings. Researchers and practitioners are encouraged to use the RE-AIM framework for beginning with the end in mind, designing for dissemination, and evaluating relevant dimensions across intervention and setting factors. Such deliberate RE-AIM application will contribute to the replicability and generalizability of planned interventions and thus yield optimal public health impact.

## Pragmatic Use of RE-AIM for Planning and Evaluation

The RE-AIM Framework can be used to direct the planning of new or ongoing interventions and systematic evaluations that include a complex interplay of individual and organizational outcomes (10). Fully employing RE-AIM can speed the translation of effective interventions in practice settings, while demonstrating impact and representativeness (9, 10). Yet, utilizing the full framework may require substantial human, data, and analytic resources that may not be available or feasibly acquired across typical clinical, community, or corporate settings (16). This is especially true in settings where decision-making may be based on a small subset of RE-AIM dimensions coupled with organizational priorities and resources.

Settings must consider the temporality of assessment for each RE-AIM dimension, which may need to occur prospectively, concurrently, and/or retrospectively to determine the impact of an initiative. While employing RE-AIM before an intervention begins is ideal to ensure careful and strategic local planning, in some cases this is not possible. Some organizational practices may be the result of opportunistic intervention, rollout from a central administrative site, innovation testing; corporate, policy, or organizational directive; or quality control and enhancement—each of which has distinct challenges in aligning the evaluation with initiative strategies.

**Figure 1:**
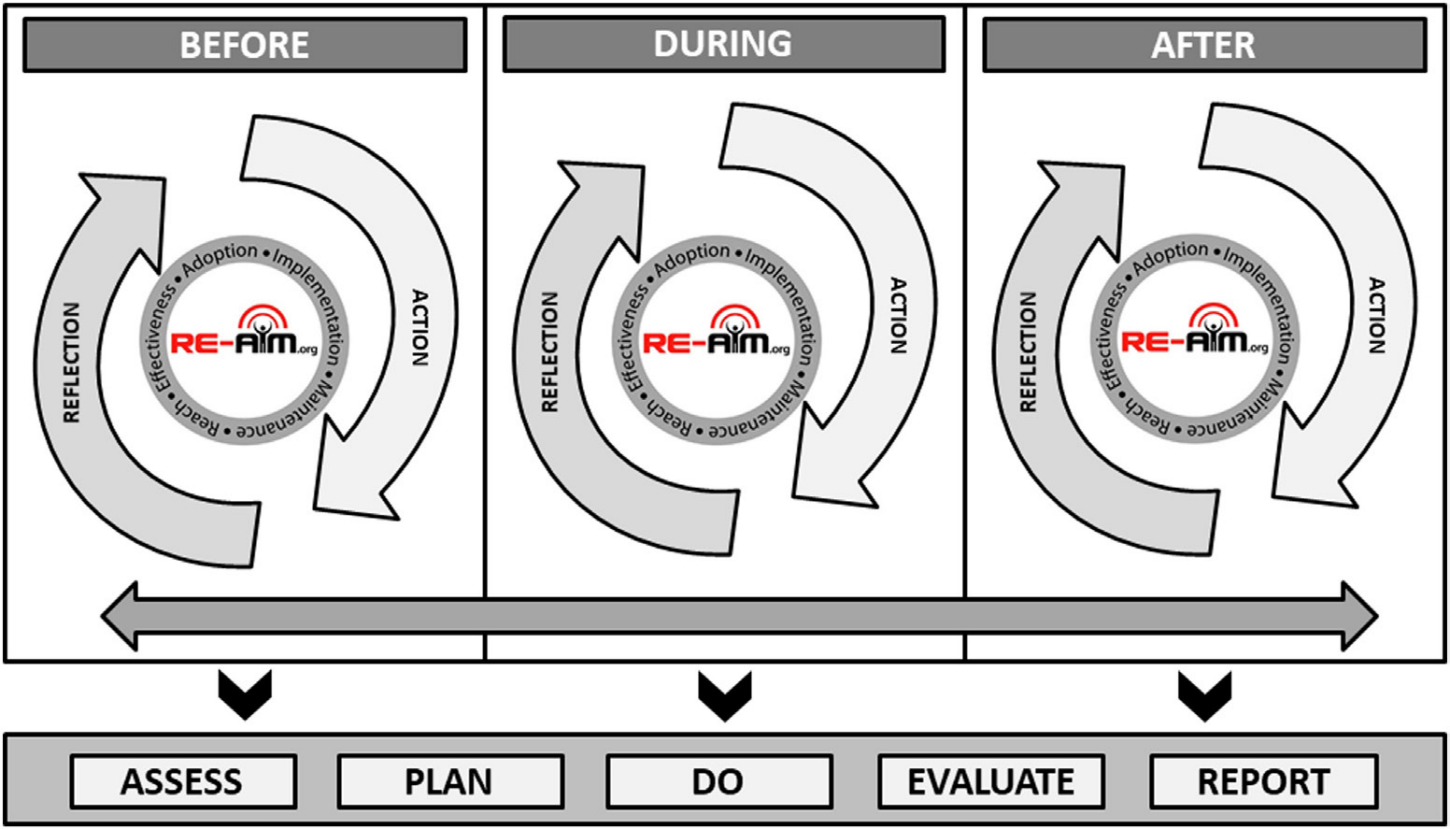
Iterative and temporal application of the RE-AIM framework.

Figure 1 illustrates the application of RE-AIM based on the starting temporal stage of an intervention or initiative, that is if the RE-AIM planning and evaluation is initiated before, during, or after an initiative has been completed. Each temporal starting point includes reflective processes in which researchers or practitioners can gather information (assess) and think critically about the relevance of each RE-AIM dimension (plan). Each stage also includes active processes where those applying RE-AIM can initiate and implement plans for interventions or initiatives (do), process gathered information based on predetermined criteria (evaluate), and engage partners and stakeholders in interpretation to support decision-making (report). The bidirectional arrow along the temporal stages indicate the iterative nature of these processes, each building upon one another to provide cumulative input for advancement and refinement based evolving priorities, challenges, and observed impacts (2, 17). The importance of Figure 1 is to address the iterative nature of applying RE-AIM in planning and evaluation and how new data are taken into consideration and used to engage in a planning and action process.

As the bidirectional arrow suggests, the end of an initiative is the beginning of another (i.e., sustained implementation, adapted implementation, or implementation of an alternative solution), thus the process is cyclical and ongoing. While it is not feasible to always employ RE-AIM before an intervention or initiative begins, this figure indicates that the process can begin at any temporal stage. At all stages, researchers and practitioners are encouraged to APDER: Assess (using relevant RE-AIM dimensions and available data); Plan (based on best science, program priorities, stakeholder and organizational values, and available resources); Do (based on predetermined plans using defined procedures/protocols and supporting appropriate adaptations as needed during implementation); Evaluate (based on criteria necessary for decision-making and iterative adjustment); and Report (to, and plan for follow-up with, key stakeholders).

The RE-AIM website^2^ hosts a planning and evaluation document, which includes prompts and considerations across all five RE-AIM dimensions by temporal stage within a project,^3^ which is also available as a supplemental table to this manuscript (see Appendix A in Supplementary Material). Selected examples of common pragmatic considerations are described below.

Engaging key stakeholders (e.g., policy makers, service delivery personnel, members from the population intended to benefit from the work) is important for guiding pragmatic evaluations using RE-AIM. Researchers and practitioners should partner with organizational decision-makers to identify the necessary information required to determine priorities, justify the need for intervention, sustain implementation, and/or broaden adoption. For example, if a strategy is delivered by a single organization with a centralized delivery infrastructure, issues related to reach and effectiveness (as well as implementation costs and sustainability) may be more relevant than adoption (18). Conversely, when attempting to scale-up or scale-out an effective intervention across a number of sites (within or across organizations), issues related to implementation quality/fidelity and adoption may be considered more important than documenting the intervention’s effectiveness in new and diverse settings (19, 20).

Pragmatically measuring RE-AIM outcomes (21) includes leveraging data already collected within the organizational setting to reduce evaluation costs and enhance local relevance. For example, imagine a health-care system will employ a multi-leveled intervention to enhance diabetes control by promoting physical activity. The intervention includes screening, brief counseling, referral to internal or external resources for physical activity. A pragmatic evaluation of this approach may include using electronic health records to assess the reach and representativeness of participants, changes in physical activity based on clinical screenings over time, and the number of referrals made (22). Based on priorities and available resources, it may be less pragmatic for the health-care system to assess patients’ use of external resources for physical activity or their actual physical activity levels. However, if a similar multi-level intervention were implemented in a community setting, accessing electronic health records may be politically, legally or cost-prohibitive, or less relevant; rather, documenting participants’ physical activity with pedometers/accelerometers and tracking facility utilization are prioritized.

Available resources for evaluation are often limited in “real world” non-academic community and clinical settings. In most settings, resources are allocated to the intervention’s delivery and management to maximize enrollment/engagement. Therefore, the pragmatic selection and use of existing measures is helpful to reduce data collection burden. However, the use of existing measures can also introduce resource needs associated with data extraction, case de-identification, and statistical analyses and data management that may exceed organizational skillsets and typical reporting procedures.

## Examples of RE-AIM in Different Settings

In this section, we provide examples of RE-AIM application in three major types of settings. In addition to these examples, Table 1 contains additional recommendations for using RE-AIM by temporal stages of an intervention (i.e., before, during, after) across clinical, community, and corporate settings. The purpose of this table is to document the consistency of topics to be considered when applying RE-AIM across settings, while highlighting the unique factors framing the contextualization of RE-AIM within settings.

**Table 1:** Examples of applying RE-AIM dimension(s) in different settings across different phases of projects.

Project stage	Clinical	Community	Corporate	Overall
**Before implementation**				

Consider the project impact on all RE-AIM dimensions and prioritize the focus for planning and evaluation	*Example*: stakeholders’ interest in intervention *reach and representativeness* within the setting			
	*Measure*: identify potentially eligible patients through electronic medical record *Considerations*: may need to conduct sensitivity analyses to determine sample size because of issues like inconsistent coding. There may be coding inconsistencies that influence the numerator or denominator, and all data may not be available for the desired study. *Prioritization*: although reach is important dimension to consider, in this example, the team priorities effect of the behavioral outcome	*Measure*: estimate and compare eligible participants to demographics using Behavioral Risk Factor Surveillance System or Census data *Considerations*: reach proportion may seem extremely small when using county-level data to determine denominator. Reach and representativeness within each delivery site, and comparisons across sites, may help understand for whom the intervention is working (or not). *Prioritization*: because the anticipated outcomes with evidence-based programs are known, the delivery of programs at multiple sites places additional emphasis on training and fidelity monitoring (to ensure outcomes are achieved).	*Measure*: identify potentially eligible participants from customers who signed up for intervention via wellness card *Considerations*: gain “buy in” from corporate leadership. Use existing corporate infrastructure to identify participants. *Prioritization*: implementation factors should be prioritized and carefully considered as they play a key role in the program’s success and ongoing sustainability. Organizations with multiple sites/locations may require local “buy in”	Attempt to keep the target population as large and diverse or representative as possible for a greater public health impact. Consider ways to enhance recruitment of those most vulnerable and most at risk. Use a team-based approach to consider which dimension is a priority for the work. Allocate resources accordingly

Determine how each dimension will be included in the project: describe, assess, and/or intervene	*Example*: decision made to *intervene* to improve adoption, *describe* effect, and *assess* implementation fidelity			
	*Intervene*: health-care organization is implementing new protocol for nursing rounds. Some clinics receive additional intervention to improve adoption of the protocol. *Describe* or measure the effect of the new rounding protocol (i.e., did it achieve outcome of interest). *Assess* the degree to which the new nurse rounding protocol was delivered consistently over time and across clinics.	*Intervene* to improve adoption rates of YMCA centers of a diabetes prevention intervention. *Describe* rates of diabetes reduction or other proximal outcomes (weight loss, physical activity improvements). *Assess* the degree to which the diabetes prevention program was delivered consistently across YMCA sites.	*Intervene* to improve adoption rates of a wellness program at a local grocery store within a national chain. *Describe* outcomes including unintended negative consequences of the wellness program. *Assess* the degree to which the wellness program was delivered consistently across grocery stores in that chain.	Avoid the publication bias for solely reporting on the effect of an intervention on the desired outcome/behavior change without describing or assessing other interventions. Consider a hybrid design when intervening or assessing both clinical/behavioral intervention as well as implementation strategy.

Develop data collection and reporting procedures and timelines for selected RE-AIM dimensions	Consider the metrics of interest and how data will be transferred. Consider if HIPPA compliance or BAA/DUA* are needed. Determine the appropriate timeline for observing outcomes (e.g., a full year of observation may be needed to see change in clinical outcomes).	Pragmatically consider what is feasible to collect based on the intended purpose of the intervention. Consider who, in what community organization, has the time and skills necessary to deliver a program. Weigh the pros and cons associated with subjective versus objective measures, primary versus secondary data, and self-reported data from participants versus administrative measures.	Consider the messages important for key stakeholders and the data that will drive such messages. Determine the time and resources needed to obtain such measures and the formats/modalities for disseminating findings to leadership and consumers.	Consider “balancing metrics” and unintended outcomes; as well as assessing and reducing potential health inequities

Engage all project staff and partners in processes to ensure transparency, equity, compliance with regulations, and support (ongoing throughout the project)	*Example*: determine appropriate stakeholders and *where, when, how, and why* they will be engaged			
	Consider structure of the clinical health-care organization and potential stakeholders including nurses, nurse assistants, physicians, patients/family, and administrators. Consider that perhaps it is not appropriate to engage patients with an electronic medical record update.	Bring together stakeholders from diverse sectors (e.g., government, academia, faith-based, aging) to allow each to vocalize their “pain points” and definitions for success. Form a comprehensive set of variables based on stakeholder priorities and use those elements to measure outcomes relevant to each stakeholder. Consider time course of putative effects	Engaging multiple employee types (leadership, different divisions/roles) in conversations about new initiatives brings a sense of ownership, which can bolster initial and ongoing support. By including multiple employee perspectives in the planning phase, the logistics about implementation and anticipated outcomes will be identified, which will increase initial adoption and the potential for long-term maintenance	Diverse perspectives allow all parties to provide feedback about processes and procedures so that a coordinated approach can be devised and executed with fidelity. Construct a logic model to understand content, activities, short- and long-term impact.

Plan for sustainability and generalizability from the outset	Consider how intervention- and assessment-components can be implemented in settings with different histories, resources, workflows Plan to communicate results with stakeholders providing guidance and align reporting of information with data needed for decision-making for sustainability	Develop a coalition or advisory board to be engaged throughout the process, including those not directly involved in the project, to identify information and resources needed to increase the likelihood of sustainability	Include staff with clinical expertise to be engaged throughout the process, including those not directly involved in the project	Design for feasibility, success, and dissemination that addresses each of RE-AIM dimensions. Design the intervention to be broadly applied within and across settings.

**DURING IMPLEMENTATION/ITERATIVE ASSESSMENT AND ADJUSTMENT**				

Monitor data periodically and at key points for each dimension (emphasis on priority dimensions)	Have brief (perhaps “automated”), ongoing data collection. Use rapid, pragmatic assessments to identify reasons for initial results	Conduct training for program delivery staff about data collection procedures including data completion and quality checks. Routinely export available data from administrative records and secondary sources to track real-time changes	Have brief “automated” ongoing data collection from routine company records. When supplementary outcome measures are used, conduct training for program delivery staff about data collection procedures including data completion and quality checks. Routinely export available data from administrative records and secondary sources to track real-time changes	Pragmatic, timely, and low-resource data collection for ongoing decision-making and engagement in the PDSA cycle over time and dimensions

Track implementation and costs as well as fidelity to core components if those are priority dimensions	Discuss and implement low burden cost assessments (interviews, tracking, observations) at key time points	Develop systems for fidelity monitoring (observation) and adherence to delivery protocol. Programs that breach fidelity are subject to additional unplanned costs (e.g., cost per participant increases if workshops are not filled to capacity)	Track implementation and variability across sites. Routinely compare outcomes across a random sample of sites as a way of identifying unanticipated fluctuations and potential protocol deviations	Real-time issues can be addressed more rapidly. Avoids type 3 error (concluding that intervention did not work when perhaps delivery was not consistent with evidence-based components)

Perform ongoing assessments of project evolution and adaptations	Probe adaptations to address each RE-AIM dimension. Track implementation and impact over time and across settings and staff	Routinely export available data from administrative records and secondary sources to track real-time progress. Regularly debrief with program deliverers and organizational partners to identify (and adapt to address) unforeseen challenges	Track implementation and impact over time and across settings and staff. Collect stories and “positive deviance” examples to inspire other settings	Need to capture real-world adaptations to systematically collect data on how, why, when, and by whom changes are being implemented in the field

Reconsider the intervention impact on (and priorities for) all RE-AIM dimensions	Use both quantitative and qualitative assessments. In applied cases, use “good enough” methods—ballpark estimates make them work when “gold standard” methods are not feasible	Assess whether the number of participants reached will enable meaningful outcomes to be observed and adjust recruitment/delivery accordingly. Discuss project progress with program deliverers, partnering organizations, and other key stakeholders regularly to ensure transparency and identify changes in priorities for the project	Assess program impact on “bottom line” and estimated return-on-investment. Discuss project progress with program deliverers, different locations, and other key stakeholders regularly to ensure transparency and identify changes in priorities for the project.	Continued discussion with stakeholders ensures that the appropriate impact is being achieved. Ongoing considerations of which dimension to intervene, describe, or assess, particularly for long-term intervention work.

Decide if adaptations are needed to address problems with outcomes on one or more RE-AIM dimensions	Pilot and then implement intervention or implementation strategy adaptations needed to improve performance, and track their impact	Assess the appropriateness of participants engaged in the intervention to determine if appropriate and equitable outcomes are observed. Depending on what is seen, there may be implications for refining participant recruitment and retention procedures	Test different intervention or implementation strategy adaptations needed to improve performance, and track their impact Track innovations	Prioritize adaptations and test their impact across dimensions (see Figure 1)

**After implementation/summative**				

Evaluate the impact on all relevant RE-AIM dimensions	Consider subgroup as well as overall effects. Consider overall impact on quality of life and patient-centered outcomes. Include balancing measures	Begin with priority dimensions and “low-hanging fruit”. Reach and implementation measures may be easily assessed, whereas adoption and maintenance may require more in-depth processes to identify	Consider subgroup effects in addition to overall outcomes. Based on findings, target intervention to streamline resources and impact	Return to RE-AIM plan and summarize accordingly. If retrospective RE-AIM evaluation, use existing tools to ensure consideration of concepts and elements within each dimension

Calculate costs and cost-effectiveness for each RE-AIM dimension	Report costs from perspective of multiple stakeholders—adopting settings; clinical team; and patients. Estimate replication costs in different settings or under different conditions	Consider the benefits of cost and cost-effectiveness in terms of expanding the initiative geographically versus scaling-up in your local area (or both). Costs may differ for new initiatives relative to those that are ongoing	Summarize return-on-investment and expected rate of return Consider how cost-saving procedures can be employed in future roll-outs	Communication and evaluation of costs contributes to generalizability of the intervention

Determine why and how observed RE-AIM results occurred	Consider using mixed methods to blend objective data (the “what”) and impressionistic data (the “why and how”) to gain a more comprehensive understanding about the context of intervention successes and challenges	Share findings with stakeholders within and external to organizations to contextualize and interpret findings. Multiple perspectives will drive decisions about impact, needed adaptations, and grand-scale dissemination (if appropriate)	Collect stories and reports about keys to success and share these at meetings, on company websites, etc.	Contribute to the understanding of the mechanisms that achieved the effect for multiple populations, settings and staff

Disseminate findings for accountability, future projects, and policy change	Base statistical findings on clinically significant findings valued by clinicians. Costs may be appropriate for leadership and health plans.	In community settings, general findings about improvements seen among participants and testimonials may be appropriate for community residents and partnering organizations	In corporate settings, metrics related to productivity and staff absenteeism may be most appropriate for leadership to assess cost–benefits of employee-level interventions. Staff outcomes and program feedback may be indicative of overall employee engagement	Determine the most appropriate format to distribute findings and which messages are most meaningful for that audience

Plan for replication in other settings based on results	Summarize lessons learned and provide guides for implementation and adaptation for different types of settings	Consider reporting venues and organizations to share results (e.g., community-based organizations, governmental agencies)	Consider issues of scalability and how to efficiently implement successful programs company-wide (with appropriate adaptations)	Develop implementation and adaptation guides for future applications and new settings

*HIPPA, health insurance portability and accountability act; BAA, business associate agreement; DUA, data use agreement.

### Clinical Health-care Setting

Esteemed professional organizations and societies (e.g., The Institute of Medicine, National Academies of Medicine, and Society of Behavioral Medicine) have called for health systems to assess key health behaviors, mental health, and social measures, and address an actionable set of social determinants of health. Leveraging these opportunities, the My Own Health Report (MOHR) consortium tested a brief, evidence-based online and interactive health risk assessment and feedback tool (MyOwnHealthReport.org). The online aid included patient-reported items on health risk behaviors, mental health, substance use, demographics, and patient preferences (23).

The MOHR project tested the interactive patient-report and feedback system in a cluster randomized trial of 18 primary care clinics across five states. RE-AIM was used to plan, adapt, and evaluate the system using a low-cost pragmatic implementation strategy. RE-AIM was used in the planning stages to develop strategies feasible for low-resource settings with patients most in need (e.g., federally qualified health centers and other diverse clinics including rural, suburban, and urban clinics). Inclusion criteria were purposively broad for clinics and patients, and time demands on patients and staff were kept to a minimum. The implementation plan involved a high degree of flexibility and allowed each clinic to recruit patients, administer the MOHR, simultaneously provide feedback, use assessment/feedback modalities, select languages (English or Spanish), and place in their clinic workflow. In terms of RE-AIM, this plan addressed reach, adoption, and implementation issues.

RE-AIM was used iteratively to monitor and adjust recruitment strategies (*r*each) and feedback and goal setting print-out delivery to patients and health-care team members (*i*mplementation). Content on print-outs were reinforced by practical webinars providing training about motivational interviewing and collaborative goal setting. The intervention was purposefully brief, low-cost (publicly available), and addressed impact (*e*ffectiveness) through standardized assessment and feedback content (23).

Results are summarized elsewhere (24), but in brief, the intervention produced high levels of reach (49% of all eligible patients, including those not contacted), adoption (18 of 30 diverse, low-income clinics approached participated), implementation (all eight risk factors assessed significantly more often in intervention patients; assessment, and print-outs delivered consistently), and effectiveness (intervention superior to randomized paired control clinics on goal setting for 6 of 8 behaviors and changes on 5 of the 8 health behavior and mental health issues). The program was not, however, *m*aintained in any of the settings following conclusion of the study.

To achieve high levels of reach, adoption, and implementation, it was necessary to allow considerable flexibility and customization about how the MOHR was delivered while keeping the content of the intervention standard (23–25). The study was conducted inexpensively and rapidly by the standards of controlled trials (25) and demonstrated use of RE-AIM for planning, adaptation, and evaluation. The lack of setting maintenance was due to the inability to integrate the intervention into the existing health records (several different EHR systems were used) and intervention costs while modest (primarily staff time) that exceeded reimbursement provided by Medicare for annual wellness exams.

### Community Setting

The RE-AIM framework was adopted in the mid-2000s for use by community-based grantees in the aging services and public health networks funded through the Administration for Community Living (26). Use of RE-AIM was part of the grant solicitation, and state grantees were expected to employ RE-AIM in their planning and evaluation of selected evidence-based interventions for managing chronic conditions. RE-AIM was chosen because of its alignment with funder goals to: “(1) develop the systems necessary to support the ongoing implementation and sustainability of evidence-based programs for older adults; (2) develop multi-sector community partnerships to enhance program accessibility and extend program capacity; (3) reach the maximum number of at-risk older adults who could benefit from the programs; and (4) deliver evidence-based programs with fidelity” (27). Consultants from the CDC Healthy Aging Research Network (28) provided technical assistance to the grantees (spanning 27 states), who were primarily aging services or public health practitioners, about how RE-AIM elements could be incorporated into their grant processes.

A questionnaire was administered to state grantees to assess the utility of the RE-AIM framework and the integration of RE-AIM elements into different planning, implementation, evaluation, and monitoring processes. Grantees reported RE-AIM was useful for planning, implementation, and evaluation and relevant for various stakeholders (e.g., evaluators, providers, community leaders, and policy makers) (26). For example, RE-AIM influenced grantee decisions about program selection, target populations, and assessment/evaluation tools. Despite the availability of technical assistance, some respondents reported difficulties in use of RE-AIM, especially adopting the framework as a whole. It was not clear if findings reflected grantees’ preferences for adopting single RE-AIM elements over the framework as a whole or if they lacked resources needed to fully assess and track all RE-AIM dimensions.

Over the past decade, RE-AIM utilization has been encouraged in other national-, state-, and local-level community-based initiatives designed to improve the healthy aging. Examples include the CDC’s Initiatives on Assuring Healthy Caregivers (29), Health Foundation of South Florida Healthy Aging Regional Collaborative (30), and the United Way Healthy Aging and Independent Living Initiative (31).

The RE-AIM framework has been valuable for helping community practitioners ask important questions during program planning, implementation, dissemination, and evaluation. However, there is often more use of and adherence to the individual RE-AIM concepts than the model as a whole, which is complicated by the changing lexicon in the field. For example, although the concepts remain consistent, recent federal aging initiatives use terms such as “scalability” and “sustainability” instead of “reach” and “maintenance.” Involvement in these aging initiatives reinforces the strong commonality between the study of aging and the RE-AIM framework: both are dynamic processes, evolving over time, and changing with the social context. For continued relevance, frameworks need to be pragmatic, fluid, and adaptable. It is a testimony to RE-AIM that its basic concepts are now mainstreamed and widely integrated into community practice.

### Corporate Setting

While theoretically as relevant and useful to corporations, the uptake of RE-AIM in corporate settings has been less frequent relative to application in clinical and community settings. Similar to other settings, corporate settings are interested in offering evidence-based programs to their consumers because programs with demonstrated *e*fficacy/*ef*fectiveness are most likely to result in positive outcomes, which ultimately satisfies key consumers and stakeholders, and sustains programs (*m*aintenance). Large corporations can have substantial *r*each because of their infrastructure and support resources (*i*mplementation) that enable rapid employment and embedding of the RE-AIM dimensions. This infrastructure allows for systematic program adoption, dissemination, and implementation supported by centralized communication channels and support staff.

The relevance and usefulness of RE-AIM in corporate settings can be demonstrated by closely examining one large US-based corporation, Walgreens. With its 8,175 locations across the US and 87 million rewards account holders, Walgreens has tremendous potential to *r*each consumers and impact public health. Even a program offered only to Walgreens’ 250,000 employees can have an impact similar to implementing a program to every resident of a moderate-size city.

With an emphasis on trust, care, and accessibility, Walgreens aims to deliver programs that improve its participants’ health and well-being. This is really no different than the goals of many non-profit, community-based organizations. What is different, however, is that Walgreens’ size and geographic dispersion makes the task of D&I somewhat daunting in terms of logistics and capital needed to initiate a system-wide intervention. Cost and perceived value are the primary reasons that health promotion programs are sustained or discontinued at the community- and corporate-level (Rhodes and Glasgow, unpublished).^4^ For example, the incentivized digital health program—*Balance Rewards for healthy choices* (BRhc)—was implemented in 2014 as a resource-efficient solution to assist Walgreens patients track health behaviors. The value of BRhc has been demonstrated by higher adherence to hypertension and diabetes medications among its users and has shown to promote physical activity among younger adults with chronic conditions (32–34). This program has a vast reach with over one million users, and the digital format of the program moderates the ongoing costs of implementation.

Based on its unique position and infrastructure (like many large corporations), Walgreens has exceeded the capability of many health care and community organizations to deliver an intervention with grand-scale reach, adoption, impact, and a maintained presence. However, substantial challenges still exist. Corporations need to value the initial investments and be convinced of adequate return-on-investment for thorough, consistent education and training of delivery staff to achieve reliable results over time (both clinical and financial). If programs are not selected, implemented, and evaluated with the utmost care, the potential patient- and organizational-level loss can be quite damaging. This is a powerful reason to advocate for expanding the application of RE-AIM within corporate settings. Utilizing RE-AIM in corporate settings can produce returns on financial investments while providing benefits to intended populations that are sustained over time.

## Discussion

This article provided a brief overview of the RE-AIM Framework and its pragmatic use for planning and evaluation while also offering recommendations to facilitate the application of RE-AIM in clinical, community, and corporate settings. Further, this article shared perspectives and lessons learned about employing RE-AIM dimensions in the planning, implementation, and evaluation phases within different settings. Due to nature and restrictions of perspective articles, we focused on limited examples of clinical, community, and corporate work. However, these detailed examples describe initial decision-making, iterative application of RE-AIM processes, and impact on public health outcomes. Similar processes can be applied in other settings for health-related outcomes. Notably, not all evaluations include all RE-AIM dimensions, and there is no right or wrong answer related to which dimensions on which to focus an evaluation. The primary dimensions deserving attention will vary by community, stakeholder and organizational priories and resources as well as the intervention settings, populations, desired outcomes, and topics. While the processes for reflection and action may differ between clinical, community, and clinical settings based on a unique set of priorities and logistics, the general considerations for applying RE-AIM remain common. To conclude, we discuss lessons learned and recommendations for how RE-AIM can be employed across settings to enhance population health in the future.

A fundamental issue across settings is whether to comprehensively apply the full RE-AIM framework or use a more limited and “strategic” approach to include only certain RE-AIM dimensions. This issue of a full versus pragmatic use of RE-AIM has recently been discussed in detail elsewhere (9, 10, 16, 22, 35), but this topic is especially relevant for applied and unfunded (or underfunded) clinical, community, and corporate non-research settings. For applied settings, the full RE-AIM Framework is best used initially at the outset and planning of a project, and then, select dimensions can be used during and after the program to guide implementation, evaluation, and/or reporting. Initial focus should focus on rough estimates of desired impact for each RE-AIM dimension, followed by decisions about: (A) which dimensions are most important for this project; (B) which dimensions should be measured given limited resources; and (C) which dimensions will be targeted for improvement. This type of pragmatic approach can engage key stakeholders through the use of existing data to determine intervention success (36). A pragmatic approach is intended to allow clinical, community, and corporate settings consider the entirety of the framework during planning, but then identify actionable RE-AIM information about the most relevant dimensions to determine if a given initiative should be abandoned, refined, sustained, scaled-up, or scaled-out (16).

Given challenges with funding (e.g., more competition to obtain limited resources) in clinical, community, and corporate settings, it is essential to consider strategies to reduce costs and leverage available resources. An interesting concept, frequent need, and important area of study is the “de-implementation” of programs and program elements that appear ineffective, too expensive, or produce unanticipated negative outcomes. Such issues need to be identified in “real time” so an intervention can be quickly modified or discontinued. The urgency of conserving costs and alleviating unnecessary spending (especially at the detriment of community well-being and health equity) highlights the need for ongoing reflection about the RE-AIM dimensions throughout the temporal stages of the intervention. As the RE-AIM framework is used to drive implementation efforts, the same framework can (and should) be used to guide and evaluate de-implementation efforts (37).

A new area of RE-AIM application involves its iterative use to provide ongoing, rapid assessments of progress, then using these results to guide program adaptations (38, 39). For example, early tracking of enrollment (*r*each) may reveal that key segments of the target population (e.g., low-income patients, those most at risk) are not participating in the intervention. Efforts can then be redirected (and tested) to improve subsequent participation rates. Although RE-AIM was initially used primarily for *post hoc* program evaluation, it was deemed useful for program planning starting in 2005 (40). Iterative uses of brief, practical measures of targeted RE-AIM dimensions are new and anticipated to grow, which warrants additional research in this area (2).

Our collective experience across clinical, community, and corporate settings indicates the need for greater attention to contextual factors. Often, the most efficient ways to assess contextual factors (the “how and why”) are qualitative or mixed-method approaches (41, 42). Such impressionistic approaches can be helpful to identify conditions under which a program is successful and reasons for such results. The Practical, Robust Implementation, and Sustainability Framework (PRISM) (43) extension of the RE-AIM model may be particularly useful for this purpose because it specifies contextual factor types that may be related to results about different RE-AIM dimensions.

The field of public health has evolved to accommodate changes in societal demographics, the environment, and impacts on the social determinants of health. In fact, such changes have caused new health-related issues and complications that spurned the creation of new fields (e.g., nutrigenomics, computational social science, behavioral economics). As fields advance, so do their need for sophisticated implementation and evaluation efforts to account for increasing complexity (e.g., big data from multiple sources/levels, nested influence and integrated variables, innovative intervention designs and statistical methodologies, systems issue and unanticipated consequences). We anticipate that the application of RE-AIM will expand to these new fields and offer a robust framework for advancing research, practice, and policy. For example, as new fields emerge and existing fields advance, the demand for multi-disciplinary collaboration grows. The RE-AIM Framework is recommended for use as a model to promote inter-professional education (using the community as the classroom) to train the next generation of scholars.

Finally, whereas much of the health promotion literature shows a publication bias toward initial effectiveness data only, using the RE-AIM framework increases the likelihood that that population-level public health impact is captured. Specifically, RE-AIM dimensions allow for the investigation of the degree to which an initiative can be *adopted* and delivered broadly, have the ability for *sustained* and consistent *implementation* at a reasonable cost *reach* large numbers of people especially those who can most benefit, produce *replicable* and *long-lasting* behavior changes. To assist with these challenges, there are RE-AIM planning and evaluation guides on the www.re-aim.org website (44).

## Conclusion

Our experience with clinical, community, and corporate initiatives highlights the importance of several factors for promoting the use of RE-AIM dimensions and methods. Calls to action include actions to: (A) recognize that technical assistance will be important for users from clinical, community, corporate, and/or academic settings to understand each RE-AIM element and how the different elements relate to one another; (B) utilize RE-AIM as a whole, but know it is acceptable to track the most relevant individual elements based on local interests and resources; and (C) give attention to common RE-AIM concepts and elements within each dimension—as well as potential measures—to bridge interventions across various clinical, community and corporate settings.

## Author Contributions

All authors contributed to the conceptualization of the manuscript and its content. All authors contributed to the full manuscript as well as reviewed and approved the final version of the manuscript.

## Conflict of Interest Statement

No financial conflicts of interest to report. All authors are members of the National Working Group on RE-AIM Planning and Evaluation Framework (www.re-aim.org).

## Footnotes

^1^www.dissemination-implementation.org.

^2^www.re-aim.org.

^3^http://re-aim.org/wp-content/uploads/2016/08/Planning-and-Evaluation-Tool.pdf.

^4^Rhodes WRD, Glasgow RE. Stakeholder perspectives on costs and resource expenditures: addressing economic issues most relevant to patients, providers and clinics. Unpublished.

## Supplementary Material

Appendix A Planning and evaluation document, which includes prompts and considerations across all five RE-AIM dimensions by temporal stage within a project (http://www.re-aim.org/resources-and-tools/self-rating-quiz/).
