# Supplementary material for: RE-AIM in Clinical, Community, and Corporate Settings: Perspectives, Strategies, and Recommendations to Enhance Public Health Impact
Source: Front Public Health. 2018 Mar 22;6:71. doi: 10.3389/fpubh.2018.00071 (PMC5874302; doi:10.3389/fpubh.2018.00071)
Supplement: Appendix A — Planning and evaluation document, which includes prompts and considerations across all five RE-AIM dimensions by temporal stage within a project (http://www.re-aim.org/resources-and-tools/self-rating-quiz/). [file Data_Sheet_1.docx]

**Planning and Evaluation Questions for Initiatives Intended to Produce Public Health Impact**

RE-AIM is a framework to help plan and evaluate different types of programs, practices, policies, and environmental changes (herein: initiative).

We developed this planning and evaluation guide using the RE-AIM framework.

The guide includes questions to help you think about things that may be important during planning or evaluating your initiative. The goal of this tool is to help you accomplish your goals.

We hope you find this guide to be helpful in an iterative way! This self-assessment exercise can be used in the planning stages, for midcourse evaluation and adjustment, and for final summative evaluation.

**Bold** questions are recommended if you have limited time or resources to collect these data.

| **Dimension** | **Example core question** | **Probes** |
| --- | --- | --- |
| **Adoption** | **What settings or organizational types are you targeting** (e.g., schools, workplaces, clinics, community setting)? | What are key characteristics of the target settings?  Who might be interested in this initiative?  How will settings hear about this?  What were characteristics of settings that did not participate (e.g., location, facilities, finances, personnel) |
|  | *** How many of these settings and organizations do you estimate will use the initiative?** | What external or environmental supports or threats are there?  How will you know if organizations used the initiative?  Who can help gather information about this? |
|  | Who will deliver the initiative (actually do the work) and do they have the skills and time? (e.g., teachers, human resources, clinicials, lay health educators, staff, delivery agents)? | What are the expertise or characteristics of those you are targeting to deliver the intervention? |
|  | *** How many of these staff (adoption agents) do you estimate will use the initiative?** | What characteristics may differ from the targeted staff and those who participate (e.g., education, time in position, training, capacity)? |
| **Reach** | **Who will benefit from the initiative?**  End-users of the program or policy initiative (e.g., students, employees, patients, kids, parents, community members) | Whom do you plan to reach in your initiative?  Please define the target population(s). |
|  | How or where will you reach them? | How will you advertise and promote the initiative?  Who needs to approve these methods? |
|  |  | How will you know if the initiative reached the intended audience and who participated? |
|  | **How will you know if those who participated are representative of the target population?** | What methods will you use to focus on health inequities?  What information is available to determine that the sample is representative of the target audience? |
| **Implementation** | **How will the initiative be delivered, including adjustments and adaptations?** | What are the key elements of the intiative that must be delivered to be successful? |
|  | **To what extent will the key aspects of the initiative be delivered as intended?** | How will you measure these data (self-report, audit, checklists)?  Describe the feasibility of these methods. |
|  | What adaptations or modifications do you think are necessary to help implement the initiative in your chosen settings? | What are likely implementation challenges you will need to overcome? |
|  | How will you know what adaptations or modifications were made during the initiative? | Who can help you keep track of modifications or adjustments made? |
|  | What are some of the possible obstacles to implementation? | Are there competing projects or programs to consider? |
|  | **What costs (inlcuding time and burden, not just money) need to be considered?** | Are these costs and resources available and reasonable to ask for (high enough priority?) |
| **Effectiveness** | **What are the most important outcomes you expect to see?** (individual-level outcomes such as more physical acivity, better quality of life, less bullying, less absenteeism, less drug use) | What is the targeted individual-level change?  How will you measure these changes?  Who will care about the outcomes?  How will you share these outcomes? |
|  | **How likely is it that your initiative will achieve its key outcomes?** | What are the biggest threats to seeing the outcomes you want? |
|  | What unintended consequences or outcomes might there be? | What has gone wrong in other similar initiatives? |
| **Maintenance** | **What will happen over the long term?** (Long-term outcomes for individuals and settings (e.g., sustained infrastructure; longer term benefits to children, employees, patients, students). |  |
|  | **Can organizations sustain the initiative over time and are there plans to leave trained staff in place?** | What infrastructure will be needed to sustain the initiative?  Is there an infrastructure and funding that will remain? |
|  | **How likely is your initiative to produce lasting effects for individual participants?** | How will individuals be delivered key program components over time? Will they stay in contact? |
|  | How will you be able to follow your initiative for an extended period of time? | How will you continue to track its success and provide ongoing feedback? |
|  | How will you get the word out about your product and lessons learned? | What easy-to-understand materials can you produce to tell others about your lessons learned? |
|  | What are likely modifcations or adaptations that will need to be made to sustain the initiative over time (e.g., lower cost, different staff, reduced intensity, different settings)? | How can you track the major changes made over time? |

**Adapted from the UPSTREAM program funded by the Colorado Health Foundation; the Evaluation Hub of the University of Colorado Department of Family Medicine; the “RE-AIM: Rate Your Plan Exercise” and the “RE-AIM: Extended Consort Diagram”; and elements from PRISM; with contributions from members of the RE-AIM workgroup.*
